# Supplementary material for: Changes in visual attentional behavior in complex regional pain syndrome: A preliminary study
Source: PLoS One. 2021 Feb 23;16(2):e0247064. doi: 10.1371/journal.pone.0247064 (PMC7901762; doi:10.1371/journal.pone.0247064)
Supplement: Attachment — Submitted filename: Response to Reviewer_ver2.docx [file pone.0247064.s003.docx]

**Response to Reviewers**

Takahiro Ushida

Multidisciplinary Pain Center, Aichi Medical University, Nagakute, Aichi, Japan

1-1 Yazako, Karimata, Nagakute, 480-1195, Japan

**RE: Manuscript ID: PONE-D-20-25338-R1**

Title: Changes in visual attentional behavior in complex regional pain syndrome: A preliminary study

Dear Dr. Smith (Academic Editor of PLOS ONE),

Thank you for granting us the chance to revise our manuscript MS ID: PONE-D-20-25338-R1, “Changes in visual attentional behavior in complex regional pain syndrome: A preliminary study”. We have responded to the comments from the Editor and have rewritten our manuscript accordingly. We hope that this version will meet with your approval.

**Signature.**

**Yukiko Shiro, Shuhei Nagai, Kazuhiro Hayashi, Shuichi Aono, Makoto Nishihara, Takahiro Ushida**

**Reviewer Comments and Response**

**Reviewer #2:**

Thank you for considering the remarks. The manuscript improved greatly.

1. Please rephrase: ll73: Depressed participants paid more attention (i.e. increased fixation duration) to sad faces significantly more than healthy controls.

**Response:** Thank you for pointing this out to us. We have rephrased the following sentence on lines 74-76 as follows.

“Depressed patients are more likely to gaze at sad faces than happy ones, compared with non-depressed subjects.”

2. l 102 and 105: The sentence “All participants provided written informed consent prior to participation in this study.“ appears twice

**Response:** Thank you for pointing this out to us. We have removed “All participants provided written informed consent prior to participation in this study.” on lines 105-106.

3. l 273 “it was reported that visual attentional bias for faces IS reduced in depressed individuals”

**Response:** Thank you for your suggestion. We have changed “it was reported that visual attentional bias for faces is reduced in depressed individuals” on line 274.

Please consider further structuring of the discussion as it remains hard to read. Consider adding subheadings for added clarity.

**Response:** In accordance with your request, we have added subheadings on lines 267, 301, 326 and 370.

Please check thoroughly for language errors!

**Response:** We have checked for language errors and made corrections where necessary. The final native check of this paper has been done by Mr. Matthew McLaughlin.
